# Menopause is a natural stage of aging: a qualitative study

**DOI:** 10.1186/s12905-020-01164-6

**Published:** 2021-02-01

**Authors:** I. M. P. S. Ilankoon, K. Samarasinghe, C. Elgán

**Affiliations:** 1 Department of Nursing and Midwifery, Faculty of Allied Health Sciences, University of Sri Jayewardenepura, Gangodawila, Nugegoda, Sri Lanka; 2 Faculty of Health Science, Kristianstad University, Kristianstad, Sweden

**Keywords:** Menopause, Experience, Qualitative study, Sri Lanka

## Abstract

**Background:**

Menopause is a biopsychosocial phenomenon encompassing the transition in a woman’s life from being fertile to infertile. Although menopause may result in extremely unpleasant physical symptoms there is evidence of a low rate of reported menopausal symptoms amongst women in Asian cultures. Women’s experiences, views, and responses to menopause which influences women’s daily life and well-being, may vary between different societies and cultures. This study aimed to explore and describe menopausal experiences among women in Sri Lanka.

**Methods:**

A qualitative exploratory research was conducted among postmenopausal women of 46–55 years of age in the western province of Sri Lanka. Individual interviews with a purposive sample of 20 women were conducted, and data analysis was done using manifest and latent content analysis.

**Results:**

The results consist of an overall theme, “Menopause is a natural stage of aging” and three categories “Entering a new stage”, “Managing menopause” and “Not the end of life” which emerged from 34 codes. The overall theme highlights that changes in menopause were experienced as a natural change in life, with health problems that are normal for this change and handled with different self-care practices. The category “Entering a new stage” describes the women becoming aware of menopause and its bodily changes. The category “Managing menopause” describes women’s experiences of being able to find their own remedies to ease the menopausal symptoms and by engaging in religious activities and focusing on interaction with people. The category, “Not the end of life” describes women's views of themselves as still valuable because menopause was experienced as a natural part of their lives.

**Conclusion:**

Women in Sri Lanka managed menopausal problem mainly on their own as they viewed the menopause as a natural stage of aging risking unnecessary suffering and failure to detect preventable complications. Enabling support groups for menopausal women and improving on their health-seeking behaviour by encouraging them to take part in screening for cervical and breast cancer would improve their condition. Further information on additional hormone therapy with a, subsequent follow-up and evaluation by community health nurses and/or midwives, would facilitate Sri Lankan women's transition to menopause.

## Background

Menopause, defined as the end of menstruation due to the loss of ovarian follicular activity, which is known to decrease in the late 30 s with complete loss in most women in the early 50 s [1] is a phenomenon of increasing concern due to an increase in life expectancy. The number of postmenopausal women worldwide is expected to reach 1.2 billion by 2030 [2]. The term ‘menopausal transition’ can be used synonymously with the term ‘peri-menopause’ [3]. The period of change in ovarian function from being fertile to becoming infertile, called menopausal transition, is a natural and inevitable change that affects all women [4]. Although, menopause is considered to be a universal phenomenon it is affected by socio-cultural norms, and women's experiences of the menopausal transition is consequently handled by women in different ways [5]. According to Meleis [6] menopause is an experience hidden within the cultural background of patriarchy for which women sacrifice their own needs including health care needs in favor of the needs of their family, consequently making menopausal transition invisible. Women have passive approach to manage symptoms using the menopausal transition due to deep roots in their cultural and traditional beliefs [7].

Nevertheless, menopause may result in extremely unpleasant physical symptoms such as atrophy of vaginal mucosa leading to vaginitis, pruritus, dyspareunia, and stenosis; genitourinary atrophy leading to urethritis, dysuria, urinary incontinence, and urinary frequency; recurrent urinary tract infections; and vasomotor symptoms such as hot flushes and night sweats. Besides gynecological health-related problems due to reduced estrogen levels, the menopausal woman has to deal with fatigue, weight gain, and emotional changes such as anxiety, sorrow, fear of illness, hypersensitivity, and irritability [8]. Women may use “natural” ways to cope with menopausal symptoms, mainly non-pharmacological methods such as diet, exercise, and herbal products that help with hot flushes [9]. There are reasons to believe that symptoms related to menopause are equally frequent regardless of geographic location, although Asian women report few menopausal symptoms [10]. Thus, it may be that cultural conditions in Asian countries make the menopausal symptoms easier to handle, or that women suffer in silence.

Sri Lanka, a middle-income South Asian country, is experiencing dramatic demographic and epidemiological changes which have led to an increase of women’s life expectancy from 74 to 78 years [11]. The mean age of menopause in Sri Lanka is around 51 years [12]. Consequently, nearly one third of a woman’s life in Sri Lanka will be post-menopausal. This will create menopause as an issue of increasing concern for health care in Sri Lanka. Further, it has been evidenced that, that joint and muscular discomfort, physical and mental exhaustion and hot flushes as the most prevalent menopausal symptoms among Sri Lankan women [13]. And women with menopausal symptoms had significantly lower quality-of-life compared with women without symptoms [13]. Further, there is an increase incidence in cardiovascular disease and osteoporosis after menopause [14]. Even though pharmacological methods, such as hormone replacement therapy (HRT), are widely advocated for the treatment of the acute symptoms of menopause, including hot flushes, vaginal dryness, night sweats, and mood swings [15], HRT is not common among women in Sri Lanka. Further, there is a lot of disinformation about various issues concerning menopausal therapy [16].

In Sri Lanka, the majority of women’s health seeking behaviors depend on their tolerance of aging or their busy lifestyle with late marriages, looking after their own children or grandchildren, household work and work in paddy fields [16]. As women in Sri Lanka are engaged in occupations or are active in their extended families by raising their grandchildren even in old age [15] it is important to strengthen the general well-being during the menopausal transition by relieving troublesome menopausal symptoms and detecting and preventing chronic diseases. Little research is done on post-menopausal women in Sri Lanka from a qualitative perspective, which motivates the aim of this study which is to explore and describe Sri Lankan women’s experiences of menopause. The knowledge provided in this research could be used to enhance care for women seeking help in menopause-related problems and provide a basis to organize health care for menopausal women based on their needs.

## Methods

### Design

An explorative and descriptive design based on an inductive qualitative approach was used to enable an understanding of postmenopausal women’s menopausal experiences. The methodology was chosen from an ontological point of view where the inquirer makes knowledge claims based primarily on constructivist perspectives emphasizing multiple meanings of individual experiences which are constructed in different socio-cultural contexts [17]. From an epistemological point of view the qualitative approach is taken by collecting stories of individuals through interviews to determine how they have personally experienced the phenomenon [18].

### Sampling and participants

The study was conducted in Boralasgamuwa Medical officer of Health (MOH) area, Colombo District in the western province of Sri Lanka. Prior to the data collection, permission was obtained from the Provincial Director of Health Services (PDHS) to conduct the study in Colombo District. Public health midwives (PHMs) who are the gate keepers of each PHM area, helped in identifying women who were within the inclusion criteria of this study from the community where they conducted the field visits. To avoid creating distance between the researcher and the women, they were informed that the researcher was a nurse who wanted to learn about their menopausal experiences.

Women meeting the inclusion criteria, which was the cessation of menstruation for at least 12 consecutive months, had not experienced any cognitive impairment and were not suffering from any acute illness such as infection or trauma by the time of interview, were asked to participate. Twenty women gave their consent to take part in the study (Table 1) after receiving written and oral information about the study.

**Table 1:** Demographic data of the participating women (n = 20)

Characteristics of participants	Frequency (n)
Age	
≥ 52 years	6
≤ 51 years	14
Religion	
Buddhist	15
Hindu	4
Christian	1
Ethnicity	
Sinhalese	16
Tamil	3
Burgher	1
Highest educational level	
Primary educational level	1
Ordinary level	9
Advance level	10
Employment status	
Employed	3
Housewife	17
Marital status	
Married	14
Separated/widowed	6
Age at menopause	
≤ 45 years	3
≥ 46 years	17

### Data collection

Individual interviews were conducted during January 2016 to July 2016 by the researcher, who is a female registered nurse and who had no prior relation to the women. A voice recorder was used to record the interviews with the consent of the participants. All interviews were conducted in the Sinhalese language and lasted between 30 and 60 min. The interviews took place in a room at the respective woman’s home, without any other person being present.

An interview guide which was developed by including specific categories of questions based on reviews of literature on women’s menopausal experiences were used. The interview guide developed for this study is provided as Additional file 1.This guide addressed the topics of experience on menopause, their understanding about menopause, and issues related to menopause. It included few socio-demographic questions such as date of birth, occupation, education, ethnicity and religion, the starting time of menopause, in order to develop a rapport before commencing the questions related to the research topic. Prompts/probes were used for clarification purposes and to get more information from the participant. The participants were asked, “Could you please tell me in your own words about your menopausal experiences?” This question was followed by probing questions such as “Could you give me an example?” “Can you elaborate on that idea?” “Could you explain that further?”.

Two pilot interviews were conducted prior to the main data collection and transcribed ad verbatim in order to refine the interview guide, Minor changes in the interview guide, such as the order of the questions and the use of probing questions, were made after the pilot interviews.

### Data analysis

The verbatim transcript was translated from Sinhalese to English by the researcher, who is fluent in both languages. The interviews were transcribed ad verbatim and the tone of voice, silence, or pauses were noted in the transcript in order to extract the authentic meaning. Transcribed interviews were analyzed by the researcher using manifest and latent content analysis according to Graneheim and Lundman [19]. This is considered an appropriate approach for analyzing interview data and interpreting its meaning in a systematic way. Content analysis focuses on the variations in respondent’ experiences and comprises descriptions of the concrete (manifest) content and interpretations of the abstract (latent) content [20].

The first step involved multiple reviews of the transcribed narrative text to obtain a comprehensive sense of the data. The second step involved extracting meaning units from the text, that is, words, sentences, or paragraphs relating to the experience of menopause. The third step involved reviewing the meaning units to ensure they contained sufficient information related to the aim of the study. In the fourth step, the meaning units were condensed by reformulating them into shorter sentences describing the experience. Condensed meaning units were then abstracted and labeled with a code (Table 2). During the condensing and coding process, the text was considered as a whole. Thirty four (34) codes were identified and in the fifth step the codes were compared based on differences and similarities and sorted into six (06) sub-categories. Similarly, sub-categories were compared based on differences and similarities and sorted into three (03) categories. Finally, an overall theme “Menopause is a natural stage of aging” emerged, that illuminated the latent content of the data. All steps were undertaken with discussions between all three authors.

**Table 2:** Example of the analysis process

Meaning unit (statements corresponding to the aim of the study)	Condensed meaning unit	Code	Sub-category	Category
Sometimes it gives me relief that I’m no longer having problems with pain and monthly bleeding. I can live more relaxed. No more bleedings and troubles with that	I feel free from pain and bleeding which gives me relief	Continue life in a more relaxed manner	Aware of menopause	Entering a new stage
I got excessive sweating. I couldn’t bear it even. I feel that I’m very weak and no strength in my body. It wasn’t reduced after treatment even. With that, I became very thin	I got excessive sweating, and I feel very weak and thin now	Experience with different body changes	Bodily changes	

### The authors’ preunderstanding

All authors are registered nurses and have a nursing perspective on the research question in focus. The first and the second authors have context-specific knowledge about Sri Lankan culture and health care, while the third author is a European researcher who had limited knowledge about the context-specific situation of menopausal women in Sri Lanka. The second and the third authors have extensive knowledge of qualitative research and content analysis.

### Rigor of the study

Trustworthiness of data and interpretation of the findings involves credibility, dependability, confirmability and transferability [21]. The first author interviewed all of the women in their natural settings to establish rapport which is to increase the credibility of the data. Dependability of the collected data was maintained by using the same pilot tested interview guide throughout the entire study. Moreover, to attain dependability and confirmability, the analysis was discussed and reviewed by all three authors and the analysis was confirmed by direct quotes from the data. However, allowing the participants to confirm the result of the data analysis would have further strengthened confirmability and thereby improved the credibility of the findings. To ensure the transferability of the findings, the authors provided detailed descriptions of the women’s experiences, including the context in their natural setting.

## Ethical approval

With reference to Codex guidelines for research (http://www.codex.uu.se/en/index.shtml), personal data including the name was not collected in order to maintain the confidentiality of the information. Before commencement of the interview, the purpose of the interview and its voluntary nature, confidentiality of the information and the use of a tape recorder, were explained to the women. Informed written consent was obtained prior to start the interview with the woman. Ethical clearance was obtained from the Ethics Review Committee of the Faculty of Medical Sciences, University of Sri Jayewardenepura and Kristianstad University, Sweden.

### Findings

The complete picture of how women in Sri Lanka experience menopause is illustrated by an overall theme Menopause is a natural stage of aging and associated with three qualitatively different categories and six subcategories (Table 3).

**Table 3:** Theme, categories, and sub-categories

Menopause is a natural stage of aging	
Category	Sub category
Entering a new stage of life	Awareness of menopause Bodily changes
Managing menopause	Self-care Religious activities Focusing on others
Not the end of life	Remaining valuable

The theme was based on that the women experienced a change of their body conceived as a natural stage of their aging process which made them view themselves as valuable as before becoming infertile. The categories are described, along with quotes to illustrate the range of experiences and ideas articulated by different participants, as indicated within brackets by the participant’s career.

### Entering a new stage of life

The category “Entering a new stage of life” meant that the women were aware that menstruation had come to a complete and permanent cessation with associated health risks. The women reported that at first they became scared, thinking that they were pregnant when their menstruation ceased for a long time.

#### Awareness of menopause

After a while, they realized that their menstruation had come to an end and that it was normal for their age, as well as for women in general, regardless of generational affiliation. The women felt relieved, as they did not have to worry anymore about pregnancies and could therefore continue life in a more relaxed manner.

I feel happy about stopping menstruation because I don’t have to be afraid of pregnancy and use any family planning method. I feel free now. (Housewife)

Sometimes it gives me relaxation that I’m no longer having problems with pain and monthly bleeding. I can live relaxed. No more bleedings and troubles with that. (Teacher).

The women expressed that they experienced bleeding, which sometimes was troublesome, after the complete and permanent cessation of menstruation. Some women had only experienced slight bleeding and did not have any problems at all, as their menstruation just suddenly stopped, while others experienced heavy bleeding with a resultant low level of hemoglobin, which eventually led to blood transfusion and removal of the womb.

I had heavy bleeding with some clots. I had this bleeding few months and I went to a doctor with my husband. Anyway I was treated for the bleeding and later my menstruation stopped completely. The doctor told me that there was a lump in the womb and removed it through a surgery. They removed the womb too. (Housewife).

According to the participants, entering menopause also involved becoming aware of menopause- related health risks such as heart problems, osteoporosis, cancer, diabetes, pressure, arthritis, and elevated cholesterol levels, which they had learned from health care personnel and from the media. The women described how to prevent some of these health problems by undergoing screening for cancer and taking calcium to prevent bone problems during menopause as well as using some type of milk products to prevent osteoporosis.

I think it is very important to take vitamins at this age. They said that our bones are not strong enough now. Anyway I don’t know why those things happen after stopping menstruation. (Housewife).

We get many health problems during this time. Some mentioned that we get bone problems. They also said that osteoporosis is common after menopause and they told we are at risk of heart attacks and cancer. (Teacher).

#### Bodily changes

The women also described menopause as a stage in life during which the body had to undergo several changes. They experienced changes such as loss of energy and body strength, weight gain or loss, excessive sweating, sleepiness, burning sensations, irritability, forgetfulness, reduced sexual appetite and pain during intercourse, thinning hair, and skin changes such as dark spots and wrinkles. They were, however, not concerned about these changes as they understood that these changes were natural, and that generations before them had dealt with the same symptoms.

Also I realized that I have wrinkles in my face. There were some dark spots too. I have seen those in other women in the same age and I thought it is the same for all. (Housewife)

I think it is the end of our energetic life as a woman. While we had menstruation, we were healthy, good looking, and had more energy. But now, it has changed. (Self-employee).

### Managing menopause

The category “Managing menopause” comprised the women’s experiences of different remedies to ease the menopausal symptoms together with religious activities and the value of feeling important to others.

#### Self-managing

According to the women, they managed menopause mostly on their own by adopting various strategies.

I think after stopping menstruation, we do not want to worry about it. It’s something natural and common to all. After having children, we do not want to worry about it know. We have a lot to do at this age. We have to think about it and do it. If we regret and stay without doing anything, there is no point of it. (Housewife).

Some of them had tried hormonal therapy for a short time, but did not find it effective. They also practiced different cultural remedies to regain lost energy during menopause.

Hathawariya (wild asparagus), gotukola (Centella asiatica). I also add curry leaves and garlic to these kanji also. These home remedies are good for health. (Housewife)

#### Religious activities

Women described how they engaged in religious activities to avoid health-related problems during menopause and to set aside menopausal symptoms. The women stated that meditation and religious Pooja (activities performed fervently by Buddhists seeking blessings) made them feel more energetic.

I chant pirith (Buddhist chanting) every night before sleep. Then I can sleep well. I do meditation also before sleep. It helps to avoid sleep problems. (Housewife)

I think that, I can avoid many problems related to menopause by doing religious activities. I believe in spiritual effects on our lives. (Self-employee).

#### Focusing on others

When health problems and bodily changes occurred due to menopausal transition, the women simply ignored them by focusing on their responsibilities to their families. By interacting with others and consequently shifting focus to other people, like helping relatives with practical matters or talking with friends, they overcame their own health problems.

I can forget about all these by attending to my work activities and my son’s studies. (Housewife)

The women emphasized that being helpful to others would help them in avoiding getting menopause-related health problems, as unselfish actions was perceived as facilitating protection against evil.

Because of the family responsibilities, I have to forget all those problems. Life is still the same as before. (Housewife)

Talking to friends about menopausal problems helped the women to manage health problems during and after menopause. It was expressed as especially important if the woman was single or had lost her spouse. The women stressed that losing a spouse or having grown-up children leave the home were common reasons for ill health and aggravated the health problems related to menopause. Still, the participants expressed an ambiguous attitude to discussing menopausal changes with others. Cessation of menstruation was especially considered as a confidential matter at an early stage of menopause, as it could be a sign of pregnancy. The women stated that their shyness of talking about menstrual matters faded with age. According to the participants, sharing menopausal experiences with other women was good practice, as it prevented women from hiding health concerns that could lead to serious health problems.

If I have a problem, I talk with my close friends. Most of us are older than 45 years, and we have similar problems. (Teacher)

There are a lot of women of my age nearby my house. We all do religious activities together. I always share my things with them. They bring me herbal drinks when I have stomach pain. They are always there to support me. (Housewife).

#### Not the end of life

The women’s experience of menopause was that it was not the end of life, but rather the end of a particular stage of aging, which necessitated overcoming and dealing with health issues and bodily changes. Many of the women mentioned how the menopause had brought about positive changes in their life pattern as they now had different needs to be accomplished before the end of their life.

#### Remaining valuable

The women viewed themselves as valuable as they continued to be engaged in different household activities and viewed these as responsibilities that they naturally assumed for their children, spouses, or other members of their families.

Although I’m getting old, I can’t stop working. I have to earn to look after my family. Sometimes I’m not well to go for work. But I can’t get leave always. (Labourer)

Even though their body had changed and they did not have the same strength as before the menopause they meant that the menopause did not prevent them from carrying out their responsibilities.

I feel that I’m losing my young life. My body is not like before. But I don’t worry about it. It’s nature. We all die. I’m also getting close to it. But we still have time to look after our children and help them with their families. (Housewife).

## Discussion

The findings of the present study on women’s experiences of menopause in Sri Lanka show that menopause was perceived as a stage of the normal aging process. This finding is consistent with findings from other research studies on women mainly derived from collectivist cultures such as those of Vietnam [3], Iran [7] and Thailand [22] for whom aging has a positive significance. Researchers argue that physiological experiences with menopause become meaningful in the shifting relations between the experiencing self and the social world [23]. The findings of the present study could therefore be explained from a sociocultural perspective, where positive attitudes about menopause is common among women in some cultures where aging comes with increased social status. This conclusion corroborates with Lazar et al. [23] study on postmenopausal women conducted using women’s views in a US-based forums on menopause and that women described menopause as a reward as it meant an end to cramps and back pain; worries about stained underwear; unwanted pregnancies and need for birth control; and worrying about what others think. Women in the current study experienced unpleasant menopausal physical symptoms as seen in the category *entering a new stage in life*, similar to other women around the world [24]. Further, they also meant that menopause had brought about positive changes in their life pattern as seen in the category *not the end of life* and that they were able to carry out their responsibilities as before the menopause as seen in the sub-category *remaining valuable.*

Coping with menopausal symptoms may differ for women in different parts of the world, as culture may affect perceptions towards menopause; which was shown by Bahri et al. [7] who found that women having a positive view towards menopause considered aging as a natural process and vice versa.

Women in this study handled their symptoms mainly through self-care that consisted of non-pharmacological methods such as the use of local herbs, and religious activities which is similar to Stanzel et al. [3] who found that Vietnamese women in Australia used self-care strategies such as exercise, dietary changes, and traditional herbs to manage menopausal symptoms due to the underlying belief that menopause is part of a natural transition in life. The women in the current study were mainly Buddhists who followed Buddhist teachings about the life cycle consisting of birth, aging, illness, and death, which may have contributed to their perceiving menopause as a natural consequence of the normal aging process. This conclusion accords with Noonil et al. [22], who studied the lived experience of Thai Buddhist women and their changing bodies in midlife. The perception of menopause as a natural component of the life cycle may however contribute in ignoring menopausal health risks or delaying necessary treatment until too late a stage. This perception may also explain why Waidyasekera et al. [13] found that women in Sri Lanka had a significantly decreased health-related quality of life due to menopausal symptoms.

Health-seeking behavior related to menopause may further be jeopardized, as taking care of family needs played an important role for the women in this study as shown in the subcategory *focusing on others*. This finding corroborates with Bahri et al. [7] who found that women in Iran were too busy to consider their menopausal symptoms due to family responsibilities. The women in the present study were aware of the physical transition from being fertile to becoming infertile, as shown in the category *entering a new stage in life.* However, rather than seeking help to overcome menopausal discomfort during the transition of menopause, the women took responsibility for their families as seen in the subcategory *remaining valuable*. Culturally, Sri Lanka is a collectivistic culture [25] with deep and strong family inter-relationships, where women traditionally have the main responsibility for looking after the home and the children. The risk of ignoring menopausal symptoms is therefore further emphasized by living in a family-oriented culture in which a woman upholds her value by giving priority to the daily care of her family members. This conclusion confirms that Sri Lankan women’s health-seeking behavior related to menopausal changes is dependent on a busy life, mainly due to family involvement [16]. It therefore seems vital to encourage Sri Lankan women to take part in screening, as they are likely to ignore serious symptoms because they prioritize family obligations instead of seeking health care for themselves.

Talking to friends about menopausal problems was a coping strategy that seemed to be useful for women in the current study as shown in the sub-category *focusing on others*. This provided the security needed not only to discuss intimate and personal matters like menopausal health issues but also to receive support to help them face losses of close relatives. The finding corroborates with the findings of Noonil et al. [22], who highlighted the importance of women sharing their experiences to overcome any changes during the menopausal transition and of support groups for middle-aged women who shared their menopausal experiences with increased self-care measures and health care interventions. Enabling support groups for menopausal women could therefore facilitate the menopausal transition for women in Sri Lanka.

The knowledge gained from this study gives rise to questions about how to improve women’s health-seeking behaviour in relation to menopausal health issues in Sri Lanka. Especially so since educational interventions with regard to menopausal issues and alternatives to ease the symptoms need to be tailored according to the prevailing sociocultural context which encompasses tolerance of aging but also a busy lifestyle related to family responsibility. Further, technological improvement with women’s increasing access to mobile phones, mobile apps reminding women of the importance of Papanicolaou (PAP) test and mammography could possibly improve women’s health-seeking behavior during menopause.

### Limitations

The transferability of the study may be limited for several reasons. The study was carried out only in one MOH area in the Western province of Sri Lanka, the majority of the participating women were housewives and only three of them were employed and finally, most of the women were Buddhists in a multicultural country.

## Conclusions

Menopause has a natural place in the aging process of women in Sri Lanka, making the symptoms easier to handle. At the same time, normalizing and ignoring menopausal symptoms, risk leading women to suffer in silence. Moreover, the detection of menopause-related health risks such as cervical and breast cancer may be ignored despite women’s awareness of the importance of attending to menopausal symptoms and related-health risks. To make consistent and reliable information available about the developmental changes and challenges associated with menopause will be beneficial to women in Sri Lanka. This could be done by implementing community-based health centers led by nurses and/or midwives who could provide information about hormonal therapy, refer to a gynaecologist, and thereafter facilitate follow-up and evaluation. Future research needs to bring together qualitative findings about the menopausal experiences of this population with quantitative data.

## Supplementary information

** Additional file 1.** Interview guide.

## Data Availability

The datasets used and/or analysed during the current study are available from the corresponding author on reasonable request.

## Abbreviations

HRT: Hormone Replacement Therapy
MOH: Medical Officer of Health
PDHS: Provincial Director of Health Services
PHMs: Public Health Midwives
WHO: World Health Organization
