# Supplementary material for: Menopause is a natural stage of aging: a qualitative study
Source: BMC Womens Health. 2021 Feb 1;21:47. doi: 10.1186/s12905-020-01164-6 (PMC7849153; doi:10.1186/s12905-020-01164-6)
Supplement: Supplementary file 1 — Additional file 1. Interview guide. [file 12905_2020_1164_MOESM1_ESM.docx]

**Additional file 01**

**Structured Interview Guide**

**Socio-demographic information**

1. Age at last birth day years
2. What is your ethnicity? a. Sinhala b. Moor

c. Indian Tamil d. Burger

e. Sri Lankan Tamil f. any others (please specify)……………………

1. What is your religion? a. Buddhism b. Hinduism

c. Roman Catholic d. Islam

e. Christian f. any others

(please specify)……………………

1. What is your highest educational qualification?
2. Primary education b. Up to O/L c. Up to A/L
3. Are you currently employed outside your house? a. Yes b. No (go to Q. No 07)

If “yes”, state your occupation………………………………………………………………….

1. Working hours? ……………………………………………………….
2. What is your marital status?

a. Currently Single b. Currently Married c. Separated/ Widow

1. Age at which you attained menopause? …………………………..

**Introduction** (10 minutes) before Interview start

Welcome and thank the participant and self-introduction.

Explain the general purpose of the study. Explain the presence and purpose of recording equipment. Address the issue of confidentiality. Inform the information is going to analyze as a group not by participants’ name.

**Purpose of interview**:

The purpose of this study is to explore more about the experiences of menopausal women and understand their expectations and knowledge about menopause and its symptoms management. This will be helpful to get deep understanding about Sri Lankan women’s perception and the different cultural practices. Findings of this research will be helpful to plan educational programme menopausal symptoms and its management.

**Interview Begins**

The questions which will be asking in semi-structured interviews:

1. What does menopause mean to you?
2. Do you consider yourself to be in the menopause?
3. How did you notice that you have come to menopause?
4. Could you please tell me in your own words about your menopausal experiences?
5. What does it mean to you by “being menopausal”?
6. What are the differences in your health/ wellbeing/ daily life before and after menopause?
7. Have you any symptoms? If yes, which symptoms?
8. How did you manage the symptoms?
9. Is there something you want to add to this interview?
10. From all that you told me today, what is the most important to you?
11. How do you feel about this interview?

Further researcher will use probes as needed. These include:

• Would you give me an example?

• Can you elaborate on that idea?

• Would you explain that further?

• I’m not sure I understand what you’re saying.

• Is there anything else?
